# Maternal obesity alters adipogenic potential and mitochondrial maximal respiration in infant mesenchymal stem cells

**DOI:** 10.3389/fendo.2026.1786389

**Published:** 2026-04-02

**Authors:** Henry A. Paz, Ying Zhong, David K. Williams, Kartik Shankar, Aline Andres, Umesh D. Wankhade

**Affiliations:** 1 Arkansas Children’s Nutrition Center, Little Rock, AR, United States; 2 Department of Pediatrics, College of Medicine, University of Arkansas for Medical Sciences, Little Rock, AR, United States; 3 Department of Biostatistics, Colleges of Medicine and Public Health, University of Arkansas for Medical Sciences, Little Rock, AR, United States; 4 Department of Pediatrics, Section of Nutrition, University of Colorado School of Medicine, Aurora, CO, United States; 5 Arkansas Children’s Research Institute, Little Rock, AR, United States

**Keywords:** adipogenic differentiation, developmental programming, maternal obesity, mitochondrial respiration, umbilical cord mesenchymal stem cells

## Abstract

**Objective:**

To assess the adipogenic potential and mitochondrial bioenergetics of umbilical cord mesenchymal stem cells (UC-MSCs) derived from infants born to mothers with divergent body mass index and to evaluate the associations between maternal BMI and adipogenic gene expression.

**Methods:**

UC-MSCs were isolated and cultured from infants born to mothers with normal weight (22.2 ± 0.3 kg/m^2^; NW-MSCs) or with overweight or obesity (29.3 ± 0.6 kg/m^2^; OW/OB-MSCs). Cells were collected at baseline (day 0) and after 7 and 14 days of differentiation to assess gene expression, protein levels, and mitochondrial respiration.

**Results:**

OW/OB-MSCs exhibited an impaired adipogenic phenotype, characterized by reduced protein levels of *Cebpa* and *Pparg* during differentiation, along with diminished mitochondrial flexibility, as evidenced by a trend toward lower maximal respiration and spare respiratory capacity compared to NW-MSCs. In addition, relationships between body mass index and expressions of *Cebpa* and *Pparg* in OW/OB-MSCs differed from NW-MSCs, particularly by day 14.

**Conclusions:**

Prenatal exposure to maternal obesity may disrupt programming of adipose precursors in offspring, impairing their adipogenic capacity and mitochondrial function, and potentially predisposing them to metabolically compromised adipose tissue later in life.

## Introduction

Maternal obesity elevates the risk of obesity in offspring, with effects that can persist from childhood into adulthood (1–3). Each 1 kg/m^2^ increase in maternal pre-pregnancy body mass index (BMI) has been associated with a 0.12% rise in neonate body fat percentage (4). This increase in adiposity affects both abdominal and non-abdominal adipose tissue in infants (5). Moreover, male infants from mothers with obesity have been reported to be three times more likely to be overweight at 1 year of age compared to female infants, which suggests sex-dependent responses to maternal BMI (6). As obesity rates continue to rise among women of reproductive age (7), it is increasingly important to investigate intrauterine programming to uncover the mechanisms driving the onset of obesity early in life. In particular, exposure to an obesogenic environment *in utero* may trigger developmental programming that predisposes offspring to long-term metabolic dysregulation, independent of postnatal influences (8–10).

Mesenchymal stem cells (MSCs) are multipotent cells capable of differentiating into various cell types of the mesodermal lineage, including adipocytes, osteocytes, and myocytes (11). Sources of perinatal MSCs include the placenta, amniotic fluid, umbilical cord (UC), and umbilical cord blood (12, 13). MSCs are emerging as a valuable tool in obesity research, as they are accessible, lack ethical concerns, and provide a unique opportunity to investigate the effects of prenatal exposures on early developmental programming (14). For instance, elevated levels of long-chain acylcarnitines, together with increased expression of lipid transport genes and indicators of oxidative stress in UC-MSCs have been related to higher adiposity at 5 months of age (15). Also, the triglyceride content of UC-MSCs has shown a positive association with fat mass percentage in children at birth, 4 to 6 months, and 4 to 6 years of age (16). Evaluation of UC-MSCs from infants born to mothers with varying BMI can help uncover the cellular-level programming effects of maternal overnutrition that contribute to obesity during early development.

Adipogenic differentiation is governed by a tightly regulated transcriptional cascade in which peroxisome proliferator-activated receptor gamma (*Pparg*) and CCAAT/enhancer-binding protein alpha (*Cebpa*) function as master regulators. *Pparg* is essential for adipocyte lineage commitment and lipid accumulation, coordinating the expression of genes involved in fatty acid uptake, triglyceride synthesis, and insulin sensitivity (17, 18). *Cebpa* acts cooperatively with *Pparg* to drive terminal adipocyte differentiation and to maintain the mature adipocyte phenotype (19, 20). Genetic or functional disruption of either gene markedly impairs adipogenesis and leads to metabolic dysfunction (21, 22). Thus, assessment of *Pparg* and *Cebpa* expression in UC-MSCs offers biologically relevant insights into early adipogenic programming and potential mechanisms linking maternal BMI to offspring metabolic risk.

In this study, we assessed differences in the expression of adipogenic gene markers and their corresponding protein levels, as well as the bioenergetic status in UC-MSCs from infants of mothers with distinct BMI. We also evaluated the associations between maternal BMI and adipogenic differentiation markers in these cells. Accordingly, the present study focused on mechanistic outcomes rather than direct phenotypic measures. We hypothesized that UC-MSCs from infants born to mothers with overweight or obesity (OW/OB-MSCs) would exhibit compromised adipogenic capacity and impaired bioenergetic response compared to those from mothers with normal weight (NW-MSCs).

## Materials and methods

### Participants

The Growing Life, Optimizing Wellness (GLOWING, NCT01131117) study enrolled female participants between 2011 and 2014 (23). The GLOWING study was a longitudinal observational study investigating the impact of maternal health before and during pregnancy on offspring growth and obesity risk. Eligible participants had a BMI between 18.5 to 35, were at least 21 years old, were in their second parity, had a singleton pregnancy, and conceived without assisted fertility treatments. Exclusion criteria included preexisting or gestational medical conditions, use of medications known to affect fetal growth, smoking, alcohol consumption, or being a professional athlete.

In the current study, we utilized a subset of participants from the GLOWING study (**Table 1**). UCs were collected from human participants at the time of delivery, with written informed consent obtained during pregnancy. The study protocol was approved by the Institutional Review Board at the University of Arkansas for Medical Sciences.

**Table 1:** Maternal and infant profiles.

Characteristics	Category^1^		
	NW (*n* = 31)	OW/OB (*n* = 33)	*P*-value^2^
Maternal^3^			
Age (years)	29.1 ± 0.7	30.0 ± 0.7	0.46
BMI before gestation week 10 (kg/m^2^)	22.2 ± 0.3	29.3 ± 0.6	<0.001
Glucose (mg/dL)			
12 weeks	79.0 ± 1.2	81.5 ± 1.0	0.12
36 weeks	78.9 ± 1.7	85.3 ± 3.2	0.08
Insulin (µU/mL)			
12 weeks	5.5 ± 0.8	7.2 ± 0.6	0.09
36 weeks	7.3 ± 0.8	18.8 ± 4.5	0.01
HOMA-IR			
12 weeks	1.1 ± 0.2	1.5 ± 0.1	0.09
36 weeks	1.5 ± 0.2	4.8 ± 1.6	0.05
Triglycerides (mg/dL)			
12 weeks	85.7 ± 5.2	107.9 ± 6.2	0.01
36 weeks	202.0 ± 15.6	206 ± 10.3	0.79
Gestational weight gain (kg)	13.4 ± 0.5	11.4 ± 1.1	0.09
Gestational age at delivery (weeks)	39.1 ± 0.2	39.3 ± 0.1	0.35
Cesarean delivery, *n* (%)	9 (29.0)	18 (54.5)	
Infant^4^			
Sex, *n* (female/male)	13/18	20/13	
Birth weight (g)	3484.3 ± 75.8	3465.5 ± 82.8	0.87
Birth length (cm)	50.8 ± 0.4	50.8 ± 0.4	0.92
FM (g)			
2 weeks	458.2 ± 17.8	473.5 ± 21.9	0.59
2 months	1054.1 ± 30.6	1085.8 ± 61.0	0.65
6 months	2141.5 ± 82.3	2317.9 ± 135.9	0.27
12 months	2655.6 ± 140.5	2706.4 ± 164.0	0.82
24 months	3270.8 ± 224.3	3375.5 ± 262.4	0.76
FM (%)			
2 weeks	12.7 ± 0.37	13.0 ± 0.4	0.60
2 months	19.8 ± 0.4	20.1 ± 0.7	0.68
6 months	28.0 ± 0.8	29.3 ± 1.1	0.34
12 months	27.1 ± 1.0	27.2 ± 1.1	0.93
24 months	26.0 ± 1.4	26.9 ± 1.7	0.71
FMI (kg/m^2^)			
2 weeks	1.7 ± 0.1	1.8 ± 0.1	0.66
2 months	3.3 ± 0.1	3.3 ± 0.2	0.74
6 months	5.0 ± 0.2	5.3 ± 0.3	0.33
12 months	4.9 ± 0.2	4.9 ± 0.3	0.89
24 months	4.4 ± 0.3	4.5 ± 0.4	0.86
FFM (g)			
2 weeks	2627.8 ± 45.7	2579.9 ± 47.6	0.47
2 months	3616.7 ± 68.8	3488.7 ± 67.5	0.19
6 months	4634.0 ± 94.5	4632.7 ± 91.3	0.99
12 months	6296.8 ± 130.8	6231.8 ± 129.6	0.73
24 months	8308.6 ± 180.9	8525.0 ± 129.5	0.34
FFM (%)			
2 weeks	73.3 ± 0.7	71.7 ± 0.6	0.08
2 months	67.9 ± 0.7	66.2 ± 0.7	0.10
6 months	60.9 ± 0.7	60.3 ± 0.8	0.56
12 months	65.2 ± 1.1	64.3 ± 0.9	0.49
24 months	67.4 ± 0.9	69.3 ± 1.1	0.19
FFMI (kg/m^2^)			
2 weeks	10.0 ± 0.1	9.8 ± 0.1	0.11
2 months	11.1 ± 0.2	10.7 ± 0.1	0.06
6 months	10.7 ± 0.2	10.7 ± 0.2	0.90
12 months	11.6 ± 0.2	11.4 ± 0.2	0.49
24 months	11.1 ± 0.2	11.3 ± 0.2	0.46

^1^NW = children born from mothers with normal weight; OW/OB = children born from mothers with overweight/obesity. Data are expressed as mean ± SEM.

^2^*P*-values are included for descriptive purpose. Independent t-test; *p* < 0.05.

^3^HOMA-IR = homeostatic model assessment-insulin resistance.

^4^FM = fat mass; FMI = fat mass index; FFM = fat-free mass; FFMI = fat-free mass index.

### Adipocyte differentiation of UC-MSCs

#### Isolation of UC-MSCs

Three-inch pieces of UC from each patient were washed three times with phosphate-buffered saline (PBS) containing 1% antibiotic-antimycotic (ABAM) (Life Technologies, Carlsbad, CA) and stored at 4 °C until processed (within 24 h of collection). UCs were sliced open longitudinally using a sterile surgical blade to expose the UC matrix, vessels were removed, and the tissue was scored horizontally multiple times to help liberate the cells. The cords were plated flat onto a 10 cm dish with the Wharton’s jelly side down in growth media consisting of low-glucose Dulbecco’s Modified Eagle’s medium (DMEM, 57%), MCDB (37%), insulin-transferrin-selenium (1x), AlbuMax (0.15 mg/mL), dexamethasone (in DMSO; 1 nM), ascorbic acid-2-phosphate (100 µM), ABAM (1%), FBS (2%), epidermal growth factor (EGF; 10 ng/mL), and platelet-derived growth factor (PDGF; 10 ng/mL) and then incubated at 37 °C and 5% CO_2_. After 7 days of incubation, the piece of tissue was removed, and patches of cells were checked until reaching 80% confluence. The cells were then split and expanded until the third passage (24).

#### Cell culture and adipocyte differentiation

UC-MSCs were thawed and maintained in growth media until reaching 80% confluence. Cells were subsequently plated at 2 × 10^5^ cells/well in 6-well plates using adipogenic media comprising of DMEM (D-glucose 1 g/L) containing 10% FBS, 1% ABAM, 1 µmol/L dexamethasone, 500 µmol/L IBMX, 5 µg/mL insulin with the addition of 60 µmol/L indomethacin (MDI-I) (24). Cells were cultured for 14 days with adipogenic media replaced every third day. Cultured UC-MSCs were collected at baseline (day 0) and at days 7 and 14 into differentiation.

### mRNA isolation and qRT-PCR

Total RNA was isolated from UC-MSCs using a combination of TRI reagent and RNeasy-mini columns (Qiagen, Valencia, CA), including on-column DNase digestion. RNA integrity (A260/A280 ratio > 1.9) and concentration were determined spectrophotometrically. 500 ng of total RNA were reverse transcribed using the iScript cDNA synthesis kit (Bio-Rad, Hercules, CA). Real-time PCR amplifications were performed using the Applied Biosystems 7500 Fast Real-Time PCR System in 10 µL final reaction volume using 2X SYBR green master mix (Applied Biosystems, Foster City, CA) with 10 ng cDNA and 0.5 µM forward and reverse primers. Gene-specific primers were designed using Primer Express Software (Applied Biosystems). Relative expression of targeted genes was normalized to the expression of 18S rRNA (25).

### Seahorse assay

UC-MSCs from days 0, 7, and 14 were plated at a density of 20,000 cells per well (n = 6/treatment) in a cell culture microplate (Agilent Seahorse XF96; Agilent, Santa Clara, CA). A Seahorse XF Cell Mito Stress test (Agilent Technologies, Cat# 103015-100) was performed 24 h after plating using the Seahorse Extracellular Flux (XFe96) Analyzer, following the manufacturer’s instructions. On the day of the assay, the cell media was replaced with 130 µL of assay media consisting of Seahorse XF DMEM freshly supplemented with 1 mM pyruvate, 2 mM glutamine, and 10 mM glucose. The cell culture microplate was incubated in a non-CO_2_ incubator at 37 °C for 40 min and then the test was conducted. During the test, cells were treated with compounds in the following order: oligomycin (2 µM), FCCP (carbonyl cyanide-4 (trifluoromethoxy) phenylhydrazone) (1.5 µM), and rotenone and antimycin A (2 µM).

### Western blot

Total cellular proteins were extracted using RIPA lysis buffer and quantified using the Pierce BCA Protein Assay Kit (ThermoFisher Scientific, Waltham, MA). For Western blots, 10 µg of protein were resolved on 4-20% SDS-PAGE gels and transferred onto PVDF membranes (Bio-Rad, Hercules, CA). Membranes were blocked in 5% milk for 1 hour and incubated overnight at 4 °C with primary antibodies against *Cebpa* (1:1000), *Pparg* (1:1000), and heat shock protein 90 (*Hsp90*; 1:1000) from Cell Signaling. *Hsp90* was used as a loading and transfer control to correct for minor lane-to-lane variability. Equal amounts of total protein were loaded per lane, and normalization was based on consistent *Hsp90* signal intensity across samples. This approach was chosen to avoid potential variability associated with cytoskeletal remodeling during mesenchymal stem cell differentiation that may affect actin-based controls (26).

Following primary antibody incubation, membranes were washed and then incubated with horseradish peroxidase-conjugated secondary antibody for 1 hour at room temperature. Protein bands were detected using chemiluminescence (SuperSignal West Femto Maximum Sensitivity Substrate; ThermoScientific, Waltham, MA) and images were captured using the Amersham Imager 600 (GE Healthcare, Chicago, IL). Densitometric analysis was performed using Image Lab software (version 6.1; Bio-Rad, Hercules, CA).

### BODIPY staining

Lipid accumulation was evaluated using boron-dipyrromethene (BODIPY) staining. Cells were stained with 2 µM BODIPY 493/503 (1:2500 dilution from 5 mM stock) for 15 min at 37 °C, and images were taken using fluorescent microscopy (Nikon Eclipse Ti2) (27). BODIPY intensity was quantified using ImageJ (version 1.54g).

### Statistical analysis

The statistical analyses were conducted using the R software version 4.3.1 (28). Comparisons between NW and OW/OB groups were performed using independent t-tests for maternal and infant characteristics, protein band intensities, and mitochondrial respiration parameters. Normality was tested using the Shapiro-Wilk test. For each gene, expression was analyzed separately using a linear mixed model with a random intercept for participant to account for repeated measures across days. The fixed-effects structure included day, category, and their interaction to test whether group differences varied over time. Models were covariate adjusted for maternal age, gestational age at delivery, gestational weight gain, infant sex, and birth weight. Estimation used maximum likelihood for comparability of fixed effects and Kenward-Roger degrees of freedom for inference. From each fitted model, we obtained marginal means (adjusted means) by day and category, and we performed day-specific contrasts. The following packages were used: lmerTest for fitting mixed models, emmeans for estimating marginal means and contrasts, and effects for plotting continuous BMI effects with bands. For regression plots, the model-based estimated values were plotted across a shared continuous BMI range, so that the regression lines represent conditional predictions from the interaction model, rather than reflecting the actual BMI distribution within each category. The panels within each figure were processed using Adobe Illustrator version 26.5.

## Results

### Maternal and infant profiles

Maternal and child characteristics are presented in **Table 1**, with comparisons included for descriptive context. As expected by study design, mothers with OW/OB exhibited higher BMI compared to mothers with NW (p < 0.001), while their maternal age (p = 0.46), gestational weight gain (p = 0.09), and gestational age at delivery (p = 0.35) were similar. Infants of mothers with NW or OW/OB had comparable birth weights (p = 0.87) and lengths (p = 0.92), as well as similar fat mass (FM, % weight) and fat-free mass (FFM, % weight) at various time points throughout the first two years (p ≥ 0.08).

### OW/OB-MSCs displayed altered expression of adipogenic markers

**Figure 1:**
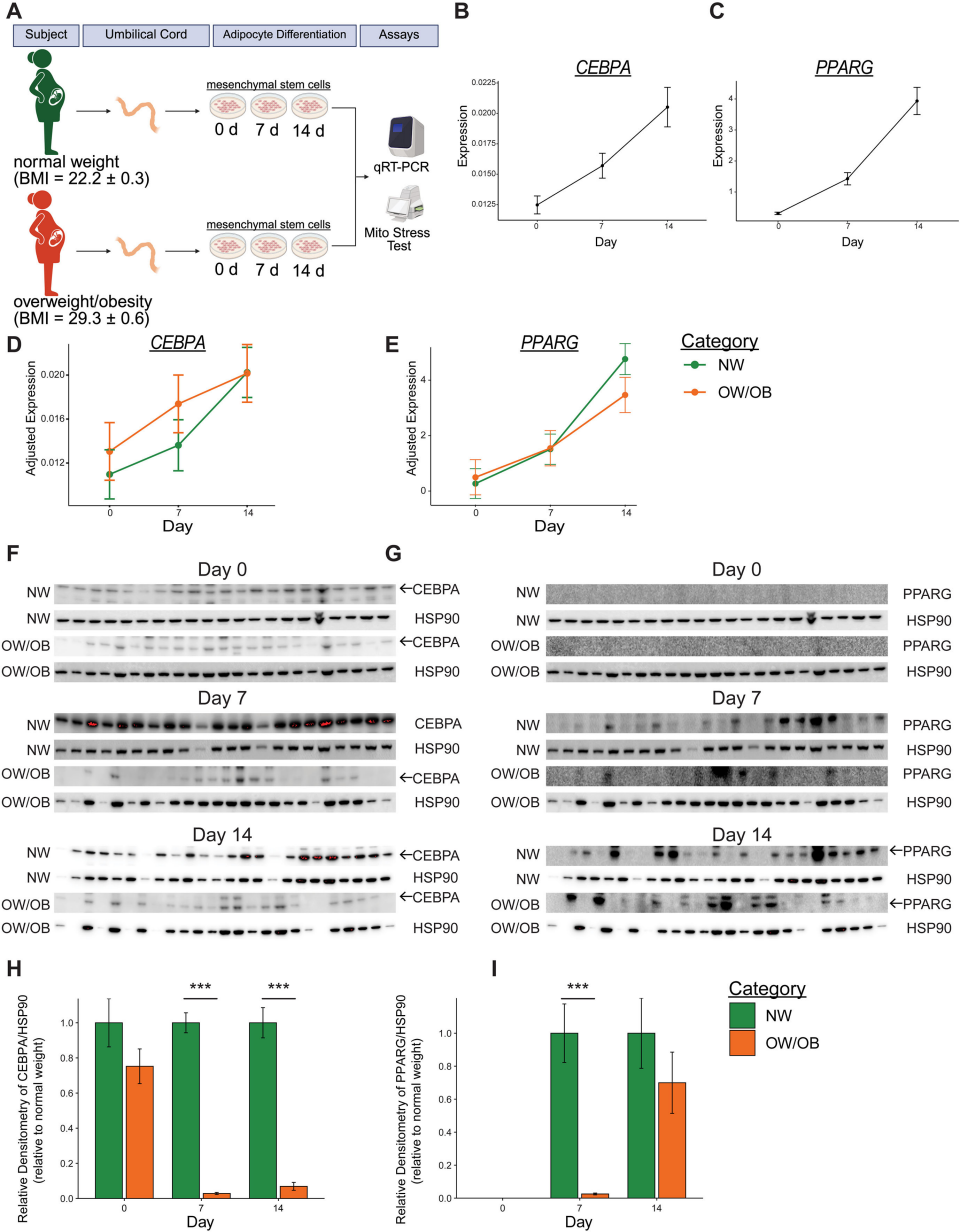
Differential expression of adipogenic markers in infant mesenchymal stem cells from mothers with divergent body mass index. **(A)** Schematic overview of the experimental scheme. Relative expression of **(B)** *Cebpa* and **(C)** *Pparg* during adipogenic differentiation of infant umbilical cord mesenchymal stem cells (UC-MSCs), normalized to 18S rRNA. Data represent pooled samples from UC-MSCs from infants born to mothers with normal weight (NW-MSCs) or mothers with overweight/obesity (OW/OB-MSCs). *n* = 30–32 per day. Adjusted mean relative expression of **(D)** *Cebpa* and **(E)** *Pparg* during differentiation in NW-MSCs and OW/OB-MSCs, normalized to 18S rRNA. *n* = 15–17 per category per day. Western blots probed for **(F)** *Cebpa* and **(G)** *Pparg*, with *Hsp90* used as a loading and transfer control. Densitometric quantitation of **(H)** *Cebpa* and **(I)** *Pparg* normalized to *Hsp90* and expressed relative to NW-MSCs. Independent t-test, ***p < 0.001.

To evaluate the impact of prenatal exposure to maternal obesity on offspring adipocyte precursors, UC-MSCs from infants born to mothers with differing BMI were assessed for adipogenic capacity and mitochondrial function, as depicted in the experimental schematic (**Figure 1A**). As anticipated, the expression of the adipogenic gene markers *Cebpa* and *Pparg* increased in UC-MSCs during differentiation (**Figures 1B, C**). For *Cebpa* expression, adjusted means from the mixed-effect model were similar between UC-MSCs from NW and OW/OB maternal groups at days 0, 7, and 14 of adipogenic differentiation (**Figure 1D**). Likewise, *Pparg* expression did not differ between UC-MSCs from both maternal groups. Nonetheless, adjusted means indicated a numerical trend toward higher *Pparg* expression in NW-MSCs compared to OW/OB-MSCs by day 14 of differentiation (**Figure 1E**). Protein levels of *Cebpa* and *Pparg* were measured to determine their correspondence with gene expression changes (**Figures 1F, G**). Unlike gene expression, *Cebpa* protein levels were 38.1-fold and 14.5-fold lower in OW/OB-MSCs compared to NW-MSCs at 7 and 14 days of differentiation, respectively (**Figure 1H**). *Pparg* protein levels also differed compared to gene expression, showing a 39.5-fold decrease in OW/OB-MSCs compared to NW-MSCs at day 7 of differentiation, with similar levels observed at day 14 (**Figure 1I**).

### Maternal BMI modulates the temporal regulation of adipogenic gene expression in infant UC-MSCs

**Figure 2:**
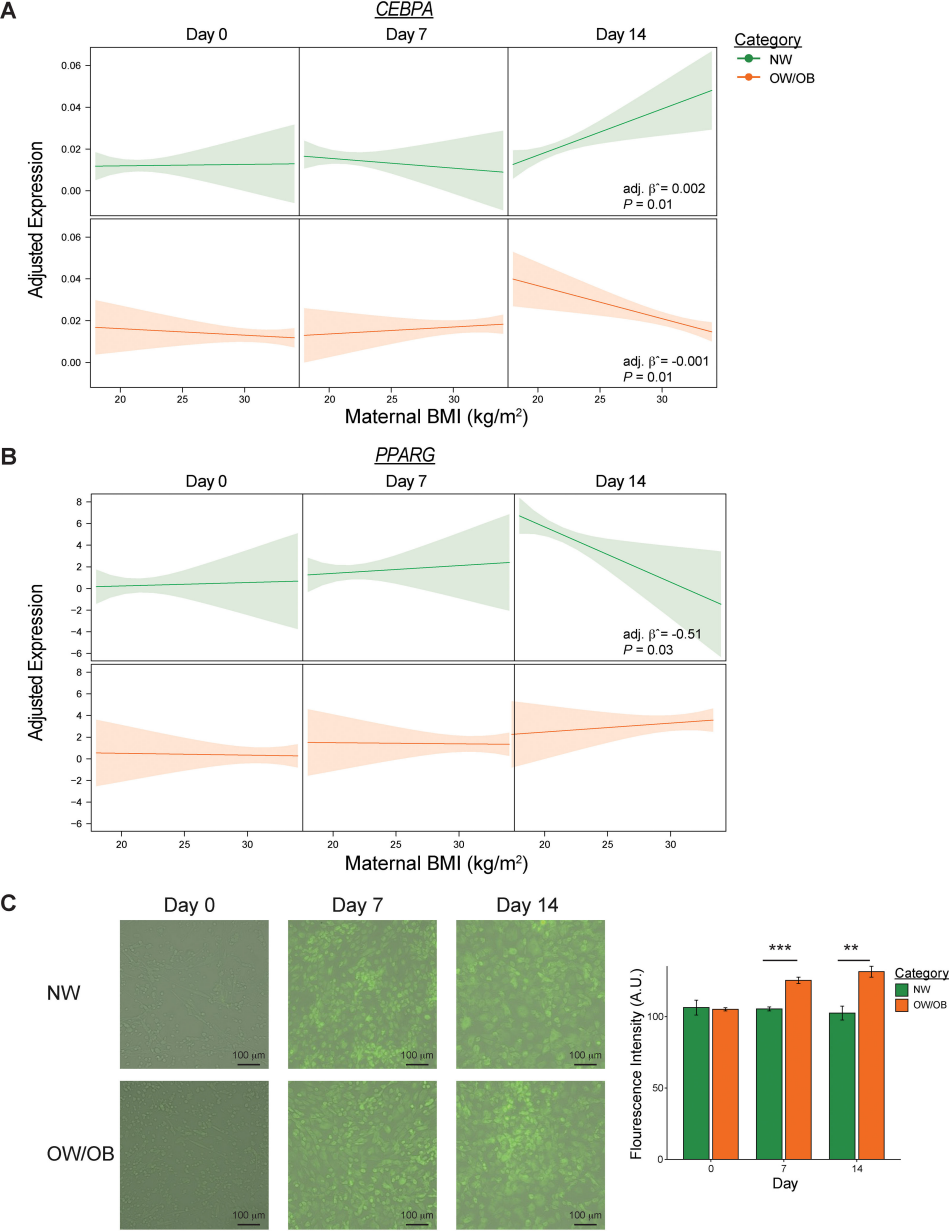
Maternal obesity alters the expression of adipogenic differentiation markers in infant mesenchymal stem cells. Associations of maternal body mass index (BMI) with **(A)** *Cebpa* and **(B)** *Pparg* expression during adipogenic differentiation of infant umbilical cord mesenchymal stem cells (UC-MSCs). For comparability across panels, model-based estimated values were plotted over a common continuous BMI range, such that regression lines reflect conditional predictions from the interaction model rather than the empirical BMI distribution within each category. **(C)** Representative images after BODIPY staining. Data in bar plot presented as as mean ± SEM (n = 10 per category per day). Independent t-test; **p < 0.01, ***p < 0.001.

To further examine the impact of maternal BMI on the adipogenic potential of UC-MSCs, we generated effect plots from mixed models (**Figures 2A, B**). The association between BMI and *Cebpa* expression among maternal groups was comparable at days 0 and 7 but diverged by day 14 of differentiation. At this time point, *Cebpa* expression increased with BMI in NW-MSCs, whereas it decreased with BMI in OW/OB-MSCs. A similar pattern was observed for *Pparg* expression, where the associations with BMI were similar at days 0 and 7 but differed at day 14 of differentiation. Specifically, *Pparg* expression decreased with BMI in NW-MSCs, while the association remained attenuated in OW/OB-MSCs. Consistent with these findings, BODIPY staining revealed progressive lipid accumulation over time in both NW- and OW/OB-MSCs. Particularly, OW/OB-MSCs displayed higher fluorescence intensity at days 7 and 14 of differentiation compared to NW-MSCs, indicating greater lipid accumulation (**Figure 2C**).

### OW/OB-MSCs exhibited a reduced capacity to respond to increased energy demands

**Figure 3:**
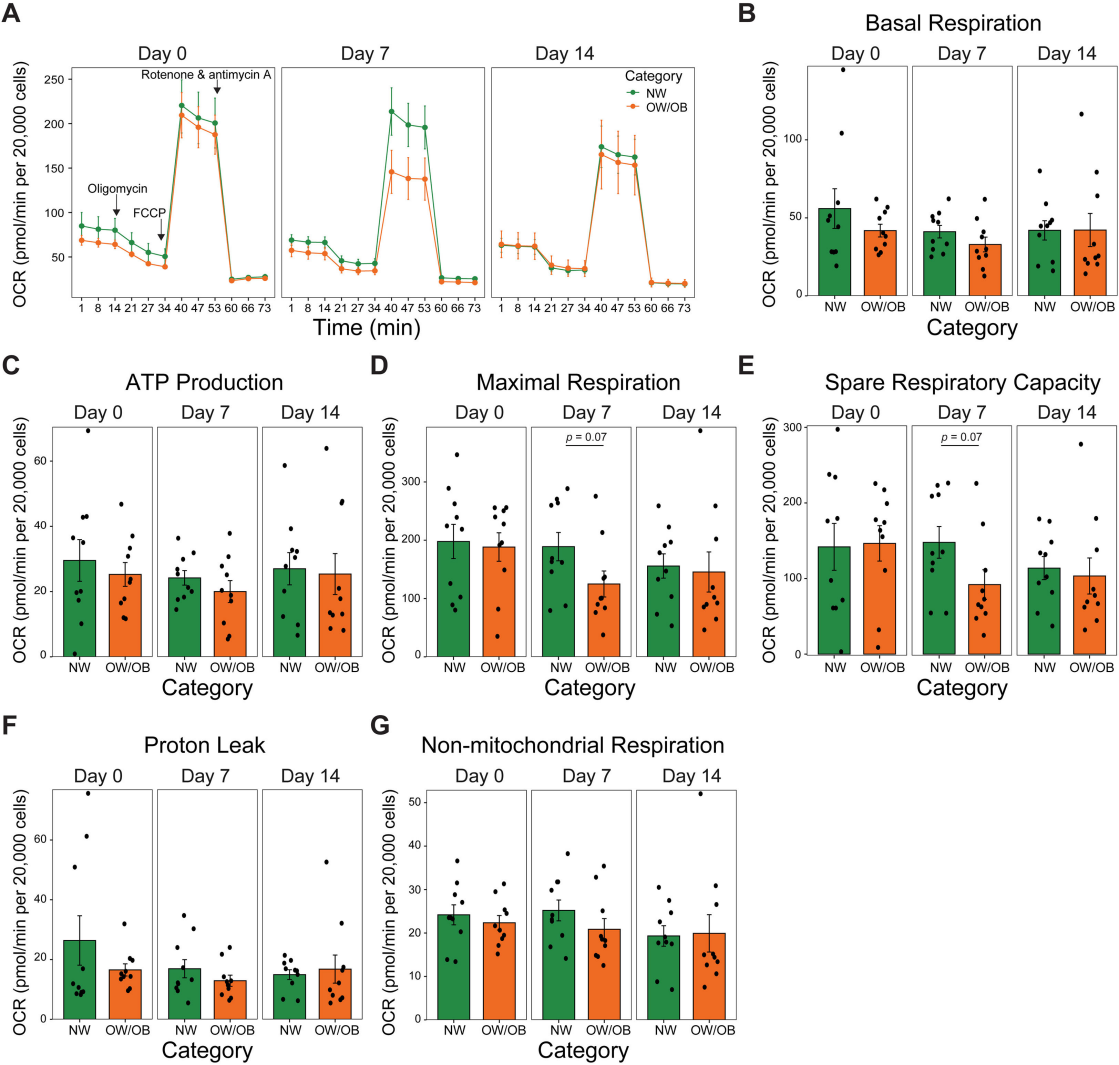
Maternal obesity decreases mitochondrial maximal respiration and spare respiratory capacity in infant mesenchymal stem cells. **(A)** Oxygen consumption rate (OCR) during mitochondrial flux assay. Bar graphs of **(B)** basal respiration, **(C)** ATP linked respiration, **(D)** maximal respiration, **(E)** spare respiratory capacity, **(F)** proton leak, and **(G)** non-mitochondrial respiration. *n* = 10 per category per day. All data presented as mean ± SEM. Independent t-test.

To evaluate bioenergetic status, UC-MSCs from both maternal groups were analyzed using the Seahorse XF Cell Mito Stress test (**Figure 3**). Mitochondrial respiration parameters, including basal respiration, ATP production, proton leak, and non-mitochondrial respiration, were similar between NW-MSCs and OW/OB-MSCs across time points. Whereas maximal respiration and spare respiratory capacity at day 7 tended to be greater (p = 0.07) in NW-MSCs compared with OW/OB-MSCs, suggesting a diminished capacity in OW/OB-MSCs to respond to increased energy requirements.

## Discussion

The rapid increase in obesity prevalence is closely linked to environmental and developmental exposures during early fetal life, with maternal obesity and related metabolic disturbances playing a central role in programming individual susceptibility to obesity (1, 29, 30). However, the mechanisms by which maternal obesity during pregnancy contributes to increased risk of obesity in offspring remain unclear. In this study, we evaluated the impact of maternal obesity on offspring predisposition to obesity by examining the phenotypic characteristics of infant UC-MSCs derived from mothers with varying BMI. We found that UC-MSCs from infants born to mothers with overweight or obesity exhibited suppressed protein levels of key adipogenic transcription factors and diminished mitochondrial bioenergetic capacity compared to UC-MSCs from NW mothers. These findings support the concept of early-life nutritional programming, whereby an adverse maternal metabolic environment may impair adipogenic potential and energy metabolism in offspring-derived adipocyte precursors.

*Pparg* and *Cebpa* are master transcription factors that act in a coordinated and mutually reinforcing manner to drive adipocyte differentiation (31, 32). In this study, protein levels of both *Cebpa* and *Pparg* were reduced during differentiation in MSCs from OW/OB mothers compared to NW mothers. Previous studies suggest that maternal obesity may induce epigenetic alterations in adipocyte precursors, thereby impairing their differentiation capacity (33–35). For instance, Boyle et al. (36) reported enhanced adipogenesis in UC-MSCs derived from obese mothers compared to NW mothers, along with a positive correlation between lipid content in adipogenic differentiating UC-MSCs and percent fat mass in infants. Taken together, these findings indicate that maternal obesity may influence adipose tissue programming in offspring, potentially predisposing them to metabolically impaired adipocytes later in life. Distinct responses were observed in gene expression and protein levels across the time points of adipogenic differentiation. Discrepancies between mRNA and protein abundance can arise from regulatory processes at multiple levels, including mRNA stability, translation efficiency, protein turnover, and post−translational modification, such that transcript levels are not always a direct proxy for protein abundance (37, 38). This discordance may reflect post−transcriptional and post−translational control mechanisms influenced by maternal obesity-associated programming. Although we detected increased lipid accumulation, indicated by BODIPY staining, in OW/OB-MSCs, these cells exhibited similar expression of adipogenic gene markers, pointing to aberrant adipogenesis or ectopic lipid accumulation rather than healthy adipocyte development. Obesity and metabolic stress have been linked to altered differentiation trajectories in MSCs populations, including incomplete or dysregulated adipogenic programming and ectopic lipid deposition in non-adipose contexts, which may manifest as stored lipids without full adipocyte maturation (39, 40).

Evaluation of the bioenergetic status of UC-MSCs during differentiation revealed a tendency towards reduced mitochondrial maximal respiration and spare respiratory capacity in OW/OB-MSCs compared to NW-MSCs. This observation is consistent with accumulating evidence that maternal obesity impairs mitochondrial function and oxidative capacity in fetal tissues, including placenta and MSCs (41, 42). Several mechanisms could underlie the reduced mitochondrial flexibility phenotype. First, epigenetic alterations on mitochondrial genes involved in ATP synthesis and fatty acid metabolism may disrupt proper mitochondrial function (43). Second, defective mitochondrial biogenesis, driven by reduced expression of key transcriptional regulators such as peroxisome proliferator-activated receptor gamma coactivator 1-alpha (*Pgc1a*), peroxisome proliferator–activated receptor gamma coactivator 1-beta (*Pcg1b*), and estrogen-related receptor alpha (*Erra*), can limit the formation of properly functional mitochondria (44). Third, heightened oxidative stress, arising from electron transport chain dysfunction and excess reactive oxygen species, can damage mitochondrial components and diminish the respiratory capacity (41). In the current study, although transient, the differences in mitochondrial bioenergetic capacity between OW/OB-MSCs and NW-MSCs during differentiation may nonetheless compromise the efficiency of downstream adipocyte function (25).

Several limitations of this study should be considered when interpreting the findings. First, UC-MSCs were studied under *in vitro* adipogenic conditions, which may not fully recapitulate the complex *in vivo* adipose tissue microenvironment or postnatal influences that shape adipocyte development. Second, our analyses were focused on key adipogenic regulators rather than employing genome-wide transcriptomic approaches. Comprehensive profiling such as RNA-Seq could identify broader gene networks and signaling pathways associated with adipogenesis and obesity susceptibility. Finally, although sex was included as a covariate, the study was not designed to evaluate sex-specific effects. Future studies incorporating larger cohorts, deeper maternal metabolic profiling, and comprehensive transcriptome-level analyses will be critical to further elucidate the molecular mechanisms linking maternal obesity to altered adipose precursor programming.

## Conclusion

Our study provides evidence that maternal overweight and obesity are associated with early alterations in adipogenic and bioenergetic profiles of UC-MSCs, which may hinder healthy adipose tissue development and increase susceptibility to obesity in offspring. Specifically, OW/OB-MSCs exhibited lower protein levels of *Cebpa* and *Pparg*, a trend toward impaired mitochondrial respiratory capacity, and distinct temporal variation in gene expression influenced by maternal BMI. These findings suggest that maternal metabolic status plays a critical role in shaping the developmental programming of adipose precursors. Understanding these *in utero* mechanisms is crucial for identifying early biomarkers of obesity risk and informing preventative interventions that prioritize maternal health before and during pregnancy to improve metabolic outcomes in the next generation. In addition, future research should explore whether these prenatal influences on adipose precursor programming differ in a sex-dependent manner, an understudied but important area for further investigation.

## Data Availability

The raw data supporting the conclusions of this article will be made available by the authors, without undue reservation.
