# Supplementary material for: Maternal obesity alters adipogenic potential and mitochondrial maximal respiration in infant mesenchymal stem cells
Source: Front Endocrinol (Lausanne). 2026 Apr 2;17:1786389. doi: 10.3389/fendo.2026.1786389 (PMC13082967; doi:10.3389/fendo.2026.1786389)
Supplement: Supplementary file 1 [file Table1.docx]

| **Primer** | **Forward** | **Reverse** |
| --- | --- | --- |
| *Cebpa* | AAGAAGTCGGTGGACAAGAACAG | TGCGCACCGCGATGT |
| *Pparg* | GAACAGATCCAGTGGTTGCAGAT | TGCAGGCTCCACTTTGATTG |
| *18S* | TCGAGGCCCTGTAATTGGAA | GCTGCTGGCACCAGACTTG |

Table S1. List of oligonucleotides used in this study.
